# Immediate Effect of Ultrasound-Guided Dry Needling on Soleus Muscle Spasticity in Stroke Survivors

**DOI:** 10.7759/cureus.62251

**Published:** 2024-06-12

**Authors:** Sanjivani N Kamble, Divya Gohil, Pravin M Pisudde, Shweta Telang-Chaudhari, Gaurang D Baxi, Tushar J Palekar

**Affiliations:** 1 Physiotherapy, Dr. D. Y. Patil College of Physiotherapy, Pune, IND; 2 Community Medicine, All India Institute of Medical Sciences, Bhatinda, IND; 3 Department of Ayurveda, Yoga & Naturopathy, Unani, Siddha, and Homeopathy (AYUSH), Maharashtra University of Health Sciences, Nashik, IND

**Keywords:** ultrasonography, modified tardieu scale, stroke, spasticity, modified modified ashworth scale, dry needling

## Abstract

Background: Dry needling (DN) is commonly used to treat various neuromuscular syndromes. It is effective in reducing spasticity in stroke and other neurological conditions. The current study explores the immediate effect of ultrasound-guided dry needling on soleus muscle spasticity and thickness in individuals with stroke.

Methods: Approval was obtained from the Institutional Sub-ethics Committee of Dr. D. Y. Patil College of Physiotherapy, Pune. The trial was registered with the Clinical Trials Registry of India. Thirty stroke survivors having soleus muscle spasticity ranging from grade 1 to 4 on the Modified Modified Ashworth Scale (MMAS) were selected. Spasticity was also assessed using the Modified Tardeau Scale (MTS) and H-reflex. Soleus muscle architecture was assessed by using ultrasonography (USG). Participants received a single session of DN for the spastic soleus muscle. Pre and immediate post-DN outcome measures were assessed.

Results: Based on USG findings, the thickness of the soleus muscle significantly increased by 2.67 mm (p<0.001) after dry needling treatment. The MMAS showed decreased spasticity by 1.47 (p<0.001) for ankle plantar flexors. A significant reduction of H-reflex values by 1.4 mV (p<0.001) was noted. The MTS also showed a significant increase in the range of ankle motion by 2.7 (p<0.001). All these indicate an immediate reduction of spasticity following DN.

Conclusion: Based on the findings of the current study, we can conclude that a single session of USG-guided DN has an immediate beneficial effect on reducing soleus muscle spasticity and increased muscle thickness in individuals with stroke.

## Introduction

Stroke is defined as the sudden loss of neurological function caused by a cessation of the blood flow to the brain, either by hemorrhage or reduced flow [1]. Stroke is globally the most common cause of functional disability [2]. The occurrence of stroke is said to be the commonest etiological factor of neurological disability in the adult population and is considered to be one of the leading causes of mortality and morbidity due to the presence of spasticity [3]. Spasticity has been defined as ‘a movement disorder characterized by an increase in tonic stretch reflexes resulting from hyper-excitability of the stretch reflex' [4]. Health-Related Quality of Life (HRQoL) is due to stroke [5]. Stroke survivors present with spasticity frequently experience secondary limb deformities and physical disability that affect their ability to perform functional activities of daily living [6].

The Modified Modified Ashworth Scale (MMAS) is used for assessing spasticity in stroke individuals. According to MMAS, grade 0 represents that there is no increase in muscle tone, whereas grade 5 shows that the affected part is rigid in flexion or extension [7]. Patients who display spasticity can seek advantages from interventions that include focal vibration in neurological rehabilitation in patients with stroke, spinal cord injury, multiple sclerosis, and parkinsonism [8]. Approaches focused on reducing spasticity include muscle stretching programs and pain management combined with pharmacological interventions [9]. Neuromuscular electrical stimulation (NMES), in combination with other modalities, helps reduce spasticity [10]. Transcutaneous Electrical Nerve Stimulation (TENS) application for 30 and 60 minutes is effective in reducing spasticity of ankle plantar flexors [11].

In addition to MMAS, the Modified Tardieu scale (MTS) is also commonly used for assessing spasticity [12]. The reliability of the components of the modified Tardieu scale is important for the accurate assessment of the dynamic component of spasticity [13]. The H reflex (Hoffmann reflex) is an electrically induced reflex similar to the spinal stretch reflex. The action potentials travel along afferent fibres until they reach and synapse alpha motor neurons. The alpha motor neurons are generated, travel along efferent fibres until they reach the neuromuscular junction, and produce a twitch response in the electromyography (EMG) [14].

Changes in the muscle architecture significantly contribute to functional impairments. Ultrasonography provides greater anatomic accuracy for examining muscle architecture in patients with spasticity [15]. It is considered to be a reproducible method for assessing muscle architecture which may guide spasticity treatment in the future.

Dry needling is effective in reducing spasticity [16,17]. Dry needling requires the insertion of thin single-filament needles into muscles, ligaments, tendons, and subcutaneous fascia in order to manage a variety of neuromuscular pain syndromes [18]. Common side effects of DN include post-needling muscle soreness, bleeding, and pain after treatment [19]. The management of stroke adults receiving dry needling may help decrease spasticity and pain and improve the range of motion [20]. Studies conducted by Yang et a. related to USG have been used to assess the muscle morphology of normal and spastic muscle architecture [15]. In order to know the significance of the effect of spasticity, this study demonstrated the changes in muscle thickness using guided USG with the application of DN.

## Materials and methods

The study design was a single group pre and post-experimental trial. The study was approved by the sub-ethics committee of Dr. D. Y. Patil College of Physiotherapy, Pune (Approval No: DYPCPT/IEC/11/2021). Informed consent was taken from all participants prior to commencement. Participants who were included in the study were diagnosed with cases of stroke, with spasticity of calf muscles ranging from grade 1 to 4 of MMAS, self-ambulatory with or without assistance, and Mini Mental Scale Examination (MMSE) intact. Participants were excluded from the study if there were any deficits in lower limb sensations, trypanophobia, blood disorders, and recurrent stroke. Pre-assessment was performed by using MMAS, MTS, H reflex and USG for soleus muscle thickness. After the intervention of USG-guided dry needling, post-assessment of muscle thickness was done immediately, followed by MMAS, MTS, and H-reflex.

Outcome measures

Spasticity Assessment

The MMAS has been shown to have good inter-rater reliability in spastic lower limb muscles [21]. According to MMAS, grade 0 represents no increase in muscle tone, grade 1 suggests that there is a slight increase in muscle tone, manifested by a catch and release at the end of the range of motion. Grade 2 represents a slight increase in muscle tone manifested by a catch, followed by minimal resistance. Grade 3 suggests that there is a marked increase in muscle tone through most of the range of motion, but the affected part can move easily. Grade 4 represents a considerable increase in muscle tone with passive movement being difficult. Grade 5 shows that the affected part is rigid in flexion or extension [7,21].

To assess spasticity by using MTS, two speeds of movement, one ‘slow’ and one ‘fast’ are used. Passive range of motion is measured using the slow speed, and is termed as R2. The fast speed is used to observe the ‘catch’ from the hyperactive stretch reflex and is termed as R1. A large variation between R1 and R2 is suggestive of spasticity. A small difference between R1 and R2 is suggestive of soft tissue changes [12].

H-reflex is a monosynaptic reflex elicited by submaximal stimulation of the tibial nerve. Patients should lie in the prone position in a comfortable manner [22]. A recording electrode is placed on the soleus muscle, a reference electrode on the Achilles tendon, and a handheld stimulating electrode is placed at the popliteal fossa, where a submaximal stimulus is applied to record a motor response. The H-reflex is elicited on submaximal stimulation and disappears on increasing stimulus [22].

Muscle Thickness Assessed by Ultrasonography

Real-time B-mode ultrasonography (USG) ​w​as used to measure muscle thickness​ using ​a 10-MHz linear transducer. This was measured with a participant in a prone position, keeping the ankle in a slightly plantarflexed position to reveal the soleus muscle [14]. Ultrasonography imaging is a feasible option for exploring muscular architecture [15].

The procedure of USG-guided dry needling

To begin with the procedure, the pre-assessment of all outcome measures was done using the Modified Modified Ashworth Scale (MMAS), followed by the Modified Tardieu Scale (MTS) using a goniometer and H-reflex by using a nerve conduction velocity machine.

Application of Procedure for Dry Needling

Before commencing needling: Prior to commencing the needling, hands were carefully sanitised and dried to maintain a sanitary environment. Hand gloves were worn to enhance hygienic measures. After the initial assessment, the taut band of spastic soleus muscle was identified by palpation. Aseptic precautions were taken for needling, and the skin area for needling was meticulously cleansed using a spirit swab to guarantee its freedom from contaminants. The area to be punctured with a needle was carefully marked with a surgical marker. USG was used to measure the pre-muscle thickness of the soleus.

During needling: There were several phases involved in finishing the needlling process. After placing the muscle in a submaximal stretch position, a 40 mm dry needling needle was used. The guiding tube and needle handle were fastened together. After that, the needle's lock was released. The guide tube and needle combination were placed over the muscle at the taut band identified by USG. The exposed handle of the needle was given a forceful, decisive tap, which caused it to pierce the skin. The guide tube was then removed. Sterile cotton was used as needed, and the needle's handle was the only part kept in touch with the shaft in order to achieve the desired depth. The segment being needled was immobilised as the local twitch response (LTR) occurred prior to neural release for 1-3 seconds. Finally, the needle was quickly removed while still in its original position, bringing an orderly and precise needling procedure to an end. These steps have been successfully completed, which is important for guaranteeing the procedure's security and correctness. After this, the muscle was repositioned to a new submaximal stretch.

After needling: The disposable needle was carefully placed in the appropriate "sharps" trash box to guarantee proper and safe disposal once the needling procedure was completed and the needle was removed from the patient. At the same time, gloves, cotton swabs, and needle coverings were thrown into an airtight, impermeable garbage container. Garbage bags and enclosed containers were sent to the biomedical waste authority. Table surfaces were also meticulously cleaned. Finally, hands were carefully washed and dried, according to the strictest hygiene regulations throughout the whole process. Post-muscle thickness was noted down immediately by USG followed by MMAS, MTS, and H reflex.

## Results

The data was analysed using Medcalc Software (version 18.2.1). Analysis was done by using the Wilcoxon Signed Rank Test (WSRT) for pre-post results. By using Wilcoxon Signed Ranks Test a statistically significant (p < 0.01) was observed for all the outcome measures. Table 1 shows the difference between pre and post mean and standard deviation values of all outcome measures used in this study.

**Table 1:** Immediate effect of dry needling on various outcome measures *Significant value (p< 0.001) Muscle Thickness: The pre mean of muscle thickness by USG was found to be 8.88 mm, which increased to 11.55 after DN. The difference was significant (p< 0.001); MMAS: The pre mean value of MMAS was 3, which reduced to 1.53 after DN. The difference was significant (p< 0.001); H reflex: The pre mean value of the amplitude of H reflex was 2.74, which reduced to 1.34 after DN. The difference was significant (p< 0.001); MTS: The pre mean value of MTS was 15.76, which increased to 18.46 after DN. The difference was significant (p< 0.001).

Outcome Measure	Pre Mean+/- SD	Post Mean+/- SD	Mean difference	Z value	P value
Ultrasound Guided Muscle Thickness	8.88+/-2.38	11.55+/-2.60	2.67+/-1.03	-4.782	<0.001*
Modified Modifies Ashworth Scale (MMAS)	3.0	1.53	1.47	-4.703	<0.001*
H-reflex	2.74+/-3.20	1.34+/-1.92	-1.4+/-1.79	-4.782	<0.001*
Modified Tardieu Scale (MTS)	15.76+/-2.22	18.46+/-2.31	2.7+/-0.09	-1.378	<0.001*

## Discussion

It is commonly recognized that dry needling is a safe, dependable, and effective way to reduce pain and stiffness in stroke patients and other neurological conditions [2]. Unfortunately, there are limited studies addressing the efficacy of dry needling treatment.

The findings of this study showed a significant increase in muscle thickness after DN intervention. Similar results following DN application were described in previous studies. Koppenhaver et al. determined the effect of DN application on the thickness of infraspinatus muscle among individuals diagnosed with subacromial pain syndrome [23]. The thickness of the superior aspect of the infraspinatus was measured by rehabilitation ultrasound imaging (RUSI), however, dry needling was applied on the inferior aspect of the muscle. Therefore, the previous study had a major limitation because the measurement occurred at a section that was distant from the site of treatment. Another case report was conducted by Cross and McMurray where the inferior aspect of the infraspinatus muscle was attempted before and after DN. The results showed an increase in infraspinatus muscle thickness immediately following DN. However, the current improvement in our study showed a significant increase in the thickness of the soleus muscle of the lower limb immediately after the application of DN [24].

Regarding the effectiveness of DN in the treatment of spasticity, the MMAS and MTS were used clinically for assessing the grade of spasticity of soleus muscle in stroke patients [7,12,13,21]. The results of our study showed a statistically significant decrease in spasticity (measured by MMAS; p < 0.001 and MTS; p < 0.001). Our findings were also in accordance with the previous study in which they showed a reduction in spasticity after 30 minutes following DN and 1 hour post-DN treatment for the upper limb [17]. A study conducted by Sanchez-Mila et al. also found a significant reduction in MMAS in both plantar flexors and dorsi flexors (p<0.001) of the foot after Bobath plus dry needling intervention [25]. However, the single session of dry needling on spasticity was not effectively determined [25]. The MMAS is still a topic of discussion because there are issues concerning validity and reliability [16,26].

When a needle is inserted into a spastic muscle, it produces a local twitch response, which creates a stretch inside the muscle. This causes a lengthening between Z-lines in the sarcomere, which in turn reduces the overlap of the contractile proteins. Additionally, dry needling reduces the firing of afferent pathways from the muscle to the spinal cord, which modifies the motor neuron's activity and tone in the stretch reflex. The alpha motor neuron and stretch reflex activity are lowered [27,28]. A comprehensive review on the effects of DN showed a reduction in muscle tone and improved gait [29]. Another study on the effects of DN on post-stroke spasticity showed a significant reduction in tone as a result of DN [30]. A case report showed similar outcomes [31]. 

Regarding the H reflex, a previous study conducted by Baraja-Vegas et al. stated that the peak amplitude was decreased when the local twitch responses were elicited during dry needling application [32]. Similar results were reported in subjects with mechanical neck pain where local twitch responses were elicited during dry needling of the upper trapezius [33]. They hypothesized that elicitation of local twitch responses during dry needling would cause disruption of dysfunctional motor end plate and modulation of motor neuron activity causing a decrease in neural pool excitability within the spinal cord [34].

Dry needling combined with other approaches was effective in reducing spasticity in both upper and lower limb muscles [23,25]. The mechanisms that can explain the effects of dry needling on spasticity are not clearly mentioned. The goal of mechanical needle insertion is to cause a localized strain that will compromise the integrity of soft tissue contractures in that area. Muscular stiffness was decreased by dry needling, which lowers muscular resistance to passive movement [2,18]. Another possibility of dry needling might help to decrease the excitability of spinal reflexes which have been suggested to have an impact on spasticity [35].

Limitations

The current study has a relatively small sample size. Also, only immediate effects have been observed. The longitudinal effects of dry needling on muscle spasticity need further exploration. Having acomparison group can add to the rigor of the study. Further studies can explore the effects of dry needling on spasticity based on the type of stroke. Also, confounding factors such as age, duration of stroke, and baseline spasticity levels can be considered in future studies, to understand their impact on the treatment outcomes.

## Conclusions

Based on the results of the current study, a single session of USG-guided DN has an immediate positive effect on reducing spasticity and muscle thickness of soleus muscle in stroke survivors. After the application of DN, a reduction in muscle tone of the spastic soleus is observed as per MMAS, MTS, and H reflex. Besides, an increase in muscle thickness is also markedly seen in the stroke population as per ultrasonography.
